# Levamisole as a Strategy Against Bacteria from Canine Otitis Externa: An In Vitro Evaluation

**DOI:** 10.3390/vetsci12070640

**Published:** 2025-07-04

**Authors:** Rodrigo F. M. Guedes, Ana C. C. F. Soares, Francisco I. F. Gomes, Alyne S. Freitas, Vinicius C. Pereira, Rossana A. Cordeiro, Marcos F. G. Rocha, José J. C. Sidrim, Giovanna R. Barbosa, Glaucia M. M. Guedes, Debora S. C. M. Castelo-Branco

**Affiliations:** 1 Course of Veterinary Medicine, Education, Sciencies and Technology Center of the Inhamuns Region, State University of Ceará, Tauá 63660-000, CE, Brazil; 2 Course of Veterinary Medicine, University of Fortaleza, Fortaleza 60811-905, CE, Brazil; anacarlacfs@gmail.com; 3 Group of Applied Medical Microbiology, Post-Graduate Program in Medical Microbiology, Federal University of Ceará, Fortaleza 60420-270, CE, Brazil; ivanilsomgomes@gmail.com (F.I.F.G.); alyne.soares_@hotmail.com (A.S.F.); viniciuscarvalhopereira99@gmail.com (V.C.P.); giovanna@ufc.br (G.R.B.); glauciademeloguedes@yahoo.com.br (G.M.M.G.); 4 Laboratory of Infectious Bioagents, Post-Graduate Program in Medical Microbiology, Federal University of Ceará, Fortaleza 60420-270, CE, Brazil; rossanacordeiro@ufc.br; 5 Post-Graduate Program in Veterinary Sciences, College of Veterinary, State University of Ceará, Fortaleza 60714-903, CE, Brazil; mfgrocha@gmail.com; 6 Specialized Medical Mycology Center, Post-Graduate Program in Medical Microbiology, Federal University of Ceará, Fortaleza 60420-270, CE, Brazil; sidrim@ufc.br

**Keywords:** canine external otitis, bacteria, biofilms, levamisole

## Abstract

Canine otitis externa is a prevalent condition, and inadequate treatment may favor the emergence of multidrug-resistant microorganisms. Drug repurposing provides an alternative approach for the management of this disease. Thus, this study aimed at assessing the antimicrobial and antibiofilm properties of levamisole against bacterial strains recovered from dogs with otitis externa as well as its influence on biofilm growth dynamics during 120 h. A total of 50 clinical bacterial isolates were subjected to analysis. Planktonic bacterial susceptibility to levamisole was assessed by broth microdilution to determine the minimum inhibitory concentration (MIC), the lowest concentration that completely inhibits bacterial growth. The activity against mature biofilms was assessed by determining the minimum biofilm eradication concentration (MBEC). The effect of levamisole on biofilm formation was evaluated at the MIC and at two subinhibitory concentrations, with daily readings recorded at 48, 72, 96, and 120 h. MICs of levamisole ranged from 0.58 to 2.34 mg/mL. Levamisole reduced the biomass of mature biofilms (*p* < 0.05), with MBEC values ranging from 1.17 to 18.75 mg/mL, and biofilm formation was significantly reduced at the MIC concentration (1.17 mg/mL) for all isolates for 120 h (*p* < 0.05). Levamisole demonstrated potential as a preventive approach against biofilm-associated bacterial otitis.

## Simple Summary

Otitis externa is an inflammation of the external ear canal with a complex and multifactorial etiology associated with predisposing, primary, and perpetuating factors in addition to secondary bacterial and fungal infections. However, the treatment of otitis is challenging when analyzing the emergence of biofilm-associated bacterial infections, which contribute to antimicrobial resistance and recurrence of the disease. In this study, levamisole was evaluated for its antimicrobial and antibiofilm effect against bacterial isolates obtained from dogs with otitis externa. The drug’s activity was assessed against bacteria both in planktonic form and biofilm-embedded. Despite its limited antimicrobial activity, levamisole was able to disrupt biofilm structure, reduce cell cohesion, and promote bacterial cell death over time. Therefore, levamisole may represent a promising strategy to prevent recurrent bacterial otitis in dogs.

## 1. Introduction

Canine otitis, one of the most common conditions encountered in dermatological clinical practice, refers to any inflammatory process affecting the ear canal of dogs [1]. The external portion comprises the auricular pinna and the vertical and horizontal ear canals [2,3], where most inflammatory processes occur [4,5]. Dogs affected by this condition may exhibit signs such as pruritus, pain, head shaking, discharge, and local erythema [1].

Predisposing factors for otitis externa, such as pendulous ears and excessive hair within the external acoustic meatus, promote cerumen accumulation and increased moisture [1,6,7]. Other factors, including allergic skin diseases, metabolic disorders, keratinization abnormalities, the presence of local tumors, and parasites, are considered primary causes, as their presence initiates the inflammatory process [5,6,7]. Additionally, inflammation-induced changes such as ear canal stenosis may lead to recurrent episodes of otitis and are classified as perpetuating factors [1].

Several etiologies of canine otitis externa lead to alterations in the microbiome of the affected anatomical site, facilitating the overgrowth of microorganisms that were previously part of the transient or commensal microbiota [8]. The main etiological agents of canine otitis externa include bacteria and yeasts, especially the species *Malassezia pachydermatis*. Among Gram-positive bacteria, *Staphylococcus* spp., β-hemolytic *Streptococcus*, and *Enterococcus* spp. are notable. Concerning Gram-negative bacteria, *Pseudomonas aeruginosa*, *Proteus* sp., and *Escherichia coli* are frequently isolated. The proliferation of microorganisms within the ear canal is considered a secondary factor in the pathophysiology of otitis externa [1,6,9,10].

It is well known that the bacteria associated with otitis externa have biofilm-forming ability, although they differ in their growth profiles. Biofilms consist of a community of microorganisms adhered to a surface or interface, embedded in a dense extracellular polymeric matrix, providing protection for the bacteria against the host’s immune system and preventing the penetration of antimicrobial agents [1,11,12].

Biofilm formation within the ear canal is an important virulence factor in canine otitis externa associated with recurrent bacterial infections. Biofilms provide protection against the antimicrobial agents used in topical treatments for otitis, consequently sustaining the proliferation of microorganisms at the anatomical site and leading to a chronic inflammatory condition [13]. Therefore, the presence of biofilm is considered a perpetuating factor in otitis externa. Bacteria such as *P*. *aeruginosa* are frequently associated with biofilm formation in the external ear canal [1,14], followed by *Staphylococcus pseudintermedius* [15].

The diagnosis of canine otitis is typically clinical. Complementary tests such as the cytology of ear secretions, combined with clinical symptoms, can help determine the severity of the condition. Microbial culture and antimicrobial susceptibility tests are rarely performed. However, it is increasingly essential to understand the isolates identified in the canal as well as their antimicrobial susceptibility. Treatment is based on the use of topical products containing glucocorticoids to reduce local inflammation, and antimicrobial agents, primarily aminoglycosides or fluoroquinolones, and azole antifungals, such as clotrimazole and miconazole [16].

The improper use of antimicrobial drugs can lead to resistance within the microbial population at the targeted anatomical sites. In the treatment of otitis externa, the empirical and recurrent use of clinically important antibiotics may result in the development of bacterial resistance. Antimicrobial resistance is an emerging and concerning issue. In response to this situation, drug repurposing has become a viable alternative for the treatment of bacterial infections [17,18].

Alternative therapies are under study with the aim of assisting conventional treatment. Compounds with no known association with bacterial resistance and capable of inhibiting biofilm formation are being tested in combination to reduce antimicrobial concentrations or to use drugs with a more restricted spectrum of action [4,5].

In this context, levamisole, a drug belonging to the imidazothiazole group, has anthelmintic and immunomodulatory properties [19]. Although previous studies have reported its antimicrobial activity against fungi [20] and its antibiofilm effects against non-fermenting Gram-negative bacteria [21], further research is needed to evaluate the antimicrobial and antibiofilm potential of levamisole against bacterial pathogens commonly associated with canine otitis externa. Therefore, this study aimed at assessing the antimicrobial and antibiofilm properties of levamisole against bacterial strains recovered from dogs with otitis externa as well as its influence on biofilm growth dynamics during 120 h.

## 2. Materials and Methods

### 2.1. Bacterial Isolates

The microorganisms in the study consist of 50 isolates of Gram-positive and Gram-negative bacteria obtained from the external ear canal of dogs diagnosed with allergic dermatitis-associated otitis externa. The study enrolled 70 dogs of different breeds (Shih Tzu, Pug, Poodle, Golden Retriever, and French Bulldog) with a history of erythroceruminous or purulent otitis externa associated with recurrent bacterial infections. Diagnosis was confirmed by otoscopic and cytological examination along with clinical signs such as erythema, edema of the pinna and external ear canal, pruritus, pain on ear palpation, head shaking, and seborrheic or purulent discharge. All animals were also diagnosed with canine atopic dermatitis based on clinical history, compatible signs, and exclusion of other pruritic conditions, following previously published criteria [22].

The isolates were selected based on their antimicrobial susceptibility profiles and their biofilm-forming capacity. These strains belong to the Group of Applied Medical Microbiology (GrAMM) of the Federal University of Ceará (UFC). Identification and antimicrobial susceptibility were performed using the MicroScan WalkAway 96 *Plus System*™ (Beckman Coulter Diagnostics, Brea, CA, USA), as described by Guedes et al. [23]. The antibacterial drugs were selected based on the antimicrobial susceptibility panel provided by the manufacturer and interpreted according to the CLSI M100-S32 [24] guidelines. The susceptibility results to some antimicrobial agents were emphasized based on the literature on systemic and topical antibiotic therapies commonly used for canine otitis externa [25]. Isolates details, susceptibility profiles, and biofilm formation are presented in Table 1.

### 2.2. Antimicrobial Susceptibility to Levamisole

The Clinical Laboratory Standards Institute (CLSI M100-S32) [24] was used as a reference to determine the minimum inhibitory concentration (MIC) of levamisole to the isolates. For the assays, levamisole hydrochloride in an injectable solution (Ripercol) at a concentration of 7.5% and a purity greater than 98% was used, obtained from a commercial source. For this, microdilution assays were performed in 96-well U-bottom plates containing Mueller–Hinton (MH) broth, in which 10 serial concentrations of levamisole were tested (0.036 to 18.75 mg/mL). Bacterial suspensions were prepared at a concentration of 5 × 10^5^ CFU/mL. Levamisole-free wells were included as growth control, and bacterium-free wells were included as a sterility control [24]. The plates were incubated at 37 °C for 24 h followed by visual reading. The tests were performed in duplicate for each strain. The MIC was defined as the lowest concentration of levamisole capable of completely inhibiting visible bacterial growth [23].

### 2.3. Cytotoxicity Assay Against MRK5

MRK5 cell line from normal human fibroblasts were seeded in 96-well plates at a density of 1 × 10^6^ cells/mL and maintained in the incubator with a 5% CO_2_ atmosphere. The cells were then exposed to levamisole at a concentration range of 0.58–18.75 mg/mL to determine the half-maximal inhibitory concentration (IC_50_) of levamisole. At the end of the treatment, Alamar Blue^®^ (Fisher Scientifics, Waltham, MA, USA) solution was added at a 1:20 concentration in RMPI culture medium without SBF, and the plate was incubated for 3 h. Then, the plates were read by fluorescence, using wavelengths of 465 nm for excitation and 540 nm for emission.

### 2.4. Susceptibility of Mature Biofilm to Levamisole

Flat-bottomed 96-well plates were used as the surface for biofilm formation, and 175 µL of Brain Heart Infusion (BHI) broth or tryptic soy broth (TSB) plus 1% glucose was used for Gram negatives and Gram positives, respectively. Then, 25 µL of bacterial inoculum at a concentration of 1.5 × 10^8^ CFU/mL was added to the wells. Subsequently, they were incubated for 48 h at 37 °C.

After this period, the culture medium was removed, and the wells were washed with 200 µL of 0.9% sterile saline solution to remove non-adherent cells. The mature biofilms were then exposed to 10 increasing concentrations of levamisole (0.036–18.75 mg/mL) in culture medium, and the plates were incubated at 37 °C for 24 h.

The metabolic activity was read by adding 20 µL of resazurin to each well and incubating for 40 min. Subsequently, a visual reading was taken to determine the minimum biofilm inhibitory concentration (MBIC), when a reduction in the biofilm’s metabolic activity was observed, shown as a partial change in the color of the resazurin, and the minimum biofilm eradication concentration (MBEC), considered to be the lowest concentration at which there was no metabolic activity and no color change was observed in the resazurin.

Biomass was then quantified by crystal violet staining technique and spectrophotometrically read at a wavelength of 490 nm (OD490) to evaluate the effect of levamisole on mature biofilms [23]. The results were compared between the wells containing biofilms exposed to different concentrations of levamisole and those of the unexposed growth control. The assays were carried out on two separate occasions.

### 2.5. Activity of Levamisole on Biofilm Formation

Biofilms were grown according to the protocol described above, and levamisole was added to the culture medium at concentrations corresponding to the MIC, MIC/2, and MIC/8 to evaluate its effect on biofilm formation. The plates were incubated at 37 °C for 48 h, and biomass was quantified using the crystal violet staining technique followed by spectrophotometric readings at 490 nm (OD490) [23]. Levamisole-free wells were included as growth control, and bacterium-free wells were included as a sterility control. Assays were performed in triplicate for each strain. The results obtained for biofilms grown in the presence of levamisole at different concentrations were compared with those obtained for the drug-free growth control.

Afterwards, the effect of levamisole on biofilm dynamics during 120 h was evaluated. For this stage, 12 isolates were used, including three bacterial strains of each of the following species: *S. pseudintermedius*, *S. schleiferi*, *P. mirabilis*, and *P. aeruginosa*, which were chosen based on their high prevalence among the evaluated dogs with otitis externa. These isolates were chosen based on their ability to form biofilms, similar susceptibility to levamisole, and similar antimicrobial susceptibility profiles.

Biofilm growth was stimulated as described in previous sections, with the addition of levamisole to the culture medium at the time of biofilm formation at concentrations corresponding to MIC, MIC/2, and MIC/8, based on the MICs obtained against each strain. Flat-bottomed microtiter plates were incubated at 37 °C for 120 h, with readings taken at 48, 72, 96, and 120 h of incubation. The culture medium was replaced at 48, 72, and 96 h of incubation. Fresh medium without levamisole was added to the growth control wells, and medium with the respective concentrations of levamisole (MIC, MIC/2, and MIC/8) was added to the test wells. Biofilm biomass was spectrophotometrically quantified at each reading point by the crystal violet staining technique [23].

Bacterium-free wells were included as sterility control, and drug-free wells were used as growth control. Tests were performed in triplicate for each strain. The biomass of levamisole-exposed growing biofilms was compared with those of the drug-free growth control. All assays were performed using culture medium with a neutral pH.

### 2.6. Assessment of Mature and Growing Biofilms Using Confocal Laser Scanning Microscopy (CLSM)

One isolate of each species, *S. pseudintermedius*, *P. aeruginosa,* and *P. mirabilis*, with good biofilm-forming ability was chosen for microscopic analysis. The biofilm growth was induced as described above, but using 24-well flat-bottomed polystyrene plates with a total volume of 1000 μL, containing 875 μL of culture medium (BHI + 1% glucose or TSB + 1% glucose for Gram-negative or Gram-positive bacteria, respectively) and 125 μL of bacterial suspension at 1.5 × 10^8^ CFU/mL. Glass slides with a diameter of 1.3 cm were added to the bottom of the wells. The plates were incubated for 48 h at 37 °C. To evaluate the effect of levamisole on the mature biofilm structure, the drug at a concentration referring to the MIC obtained against each isolate was added after 48 h of biofilm growth, and the plate was reincubated for a further 24 h. To determine the effect of levamisole on biofilm growth, it was added at a concentration referring to the MIC obtained against each isolate, at the time when biofilm growth was induced, and plates were incubated for 48 h.

The glass slides were washed and stained with 100 μL of FilmTracer LIVE/DEAD Biofilm Viability™ (Thermo Fisher Scientific, Waltham, MA, USA). A Nikon C2 confocal microscope (Nikon, Tokyo, Japan) was used at 488 nm for the detection of SYTO9, which crosses intact membranes and identifies live cells, and at 561 nm for the detection of propidium iodide, which identifies only dead cells or cells with damaged membranes. The three-dimensional images (Z-stack) of 7 fields/glass slides were acquired with the Nikon Eclipse Ti camera and processed with the NIS elements AR software 2.0. The images were analyzed with the COMSTAT2 software, associated with ImageJ 1.5i, to calculate the maximum thickness, the average biomass thickness (μm), and the surface/volume ratio (μm^2^/μm^3^) [23].

### 2.7. Statistical Analysis

The tests were performed in replicates at two separate time points. The results were subjected to normality tests. When data did not present a normal distribution, non-parametric analysis was carried out. For the comparison between MIC and MBEC, the Mann–Whitney test was applied. In the evaluation of biofilms, either Student’s *t* test for paired samples for normally distributed data or Wilcoxon’s test for asymmetric data was performed. Hypotheses were tested at a significance level of 5%. The statistical analysis was performed using GraphPad Prism 9.0.

## 3. Results

### 3.1. Minimum Inhibitory Concentrations (MICs) of Levamisole and Cytotoxicity Assays

The MICs of levamisole against the tested isolates ranged from 0.58 to 2.34 mg/mL. For Gram-positive species, MICs reached the minimum value of 0.58 mg/mL; however, for the three *E. faecalis* strains, levamisole MICs were 2.34 mg/mL. On the other hand, MICs for Gram-negative species ranged from 1.17 to 2.34 mg/mL. Overall, Gram-negative species exhibited higher minimum inhibitory concentrations (MICs) compared with Gram-positive species (*p* < 0.05) (Table 2). Regarding the cytotoxicity test, levamisole IC50 against MRK5 was 1.2 mg/mL (CI95: 0.82–1.76).

### 3.2. Minimum Biofilm Inhibitory and Eradication Concentrations of Levamisole

Levamisole MBICs against Gram-positive and Gram-negative bacteria used in this study ranged from 2.34 to 18.75 mg/mL, with a modal value of 9.37 mg/mL observed in 18/50 (36%) of isolates. MBECs ranged from 4.68 to 18.75 mg/mL against Gram-positive and Gram-negative isolates; however, they could not be determined (>18.75 mg/mL) against 28/50 (56%) of isolates (Table 2).

Levamisole MBECs ranged from 4.68 to > 18.75 mg/mL and were up to 64 times higher than the MICs against *S. pseudintermedius*, *S. aureus*, and *S. auricularis*; up to 32 times higher than the MICs against *P. mirabilis* and *E. coli*; and up to 16 times higher than the MICs against *P. aeruginosa* and *E*. *faecalis* (Table 2). Overall, levamisole MBECs were significantly higher (*p* < 0.05) than the MICs.

**Figure 1:**
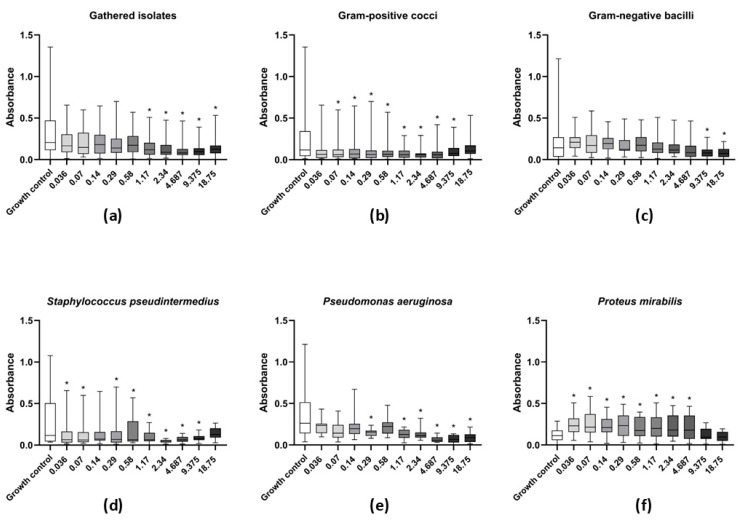
Activity of levamisole against mature biofilms of bacteria recovered from dogs with otitis externa. Data expressed as mean ± standard deviation of biomass absorbance measured by crystal violet. (**a**) General profile of levamisole activity against mature biofilms of all tested isolates; (**b**,**c**) levamisole activity against Gram-positive cocci or Gram-negative bacilli, respectively; (**d**–**f**) levamisole activity against *Staphylococcus pseudintermedius* (*n* = 11), *Pseudomonas aeruginosa* (*n* = 11), and *Proteus mirabilis* (*n* = 12). * *p* < 0.05 indicates significant differences compared with the unexposed growth control.

A significant reduction in the biomass of mature biofilms was observed for all tested bacterial isolates (*n* = 50) at levamisole concentrations ranging from 1.17 to 18.75 mg/mL (*p* < 0.05) when compared with the drug-free growth control (Figure 1a). Levamisole reduced mature biofilm biomass of Gram-positive isolates at concentrations ranging from 0.07 to 9.37 mg/mL (*p* < 0.05) (Figure 1b). Mature biofilms of *S. pseudintermedius* showed significant biomass reduction after exposure to levamisole at concentrations ranging from 0.03 to 9.37 mg/mL (*p* < 0.05) (Figure 1d).

**Figure 2:**
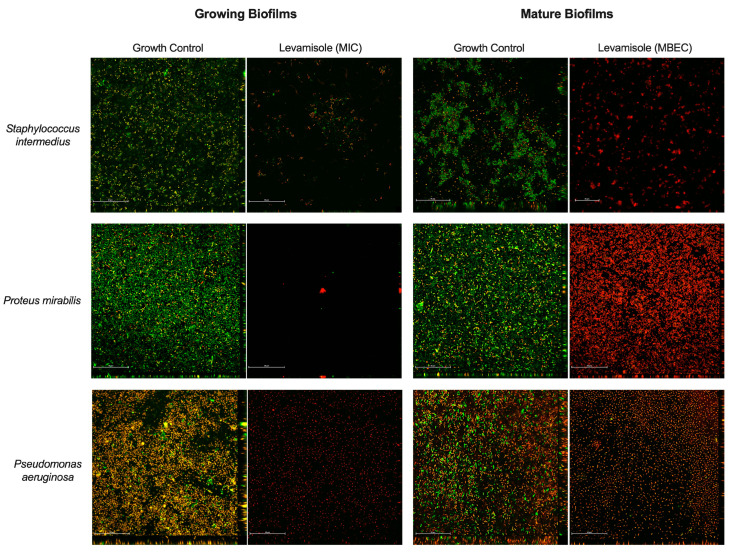
Analyses of growing and mature biofilms of *Staphylococcus pseudintermedius*, *Proteus mirabilis,* and *Pseudomonas aeruginosa* exposed to levamisole by confocal laser scanning microscopy. Exposure of biofilms to levamisole resulted in lower viable cell density compared with controls both during growth and in mature biofilms formed after 48 h. Confocal microscopy with Live/Dead™ fluorescent dye was employed. Emission at 488 nm revealed the SYTO9 fluorophore, which marks live cells (green), while emission at 561 nm detected dead or damaged cells (red). Visualization under 600× magnification. Scale: 50 µm.

**Figure 3:**
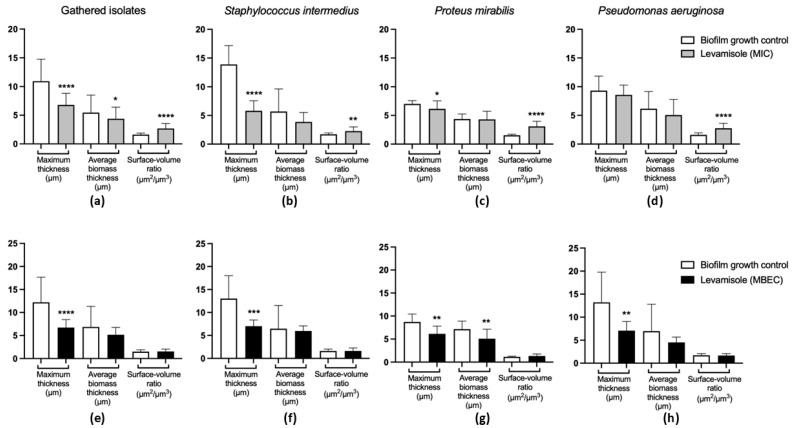
Quantitative evaluation of images obtained by confocal laser scanning microscopic of *Staphylococcus pseudintermedius* (*n* = 1), *Proteus mirabilis* (*n* = 1), and *Pseudomonas aeruginosa* (*n* = 1) growing and mature biofilms treated with levamisole. (**a**) General activity of levamisole at the minimum inhibitory concentration (MIC) on the structural organization of growing biofilms of the three isolates analyzed. (**b**–**d**) Activity of levamisole (MIC) on the morphology of growing biofilms of *S. pseudintermedius*, *P. mirabilis,* and *P. aeruginosa*, respectively. (**e**) General activity of levamisole at the minimum biofilm eradication concentration (MBEC) on the structural organization of mature biofilms of the three isolates analyzed. (**f**–**h**) Activity of levamisole (MBEC) on the morphology of mature biofilms of *S. pseudintermedius*, *P. mirabilis,* and *P. aeruginosa*, respectively. Levamisole exposure resulted in reduced maximum and biomass average thickness and increased surface-to-volume ratio, indicating disorganization of biofilm structure, with lower cohesion among cells. Biofilms were stained with Live/Dead™ dye, and Z-stack images were quantitatively analyzed using COMSTAT. Data are shown as mean ± standard deviation. * *p* < 0.05; ** *p* < 0.01; *** *p* < 0.001; and **** *p* < 0.0001 indicate statistically significant differences compared with unexposed growth controls.

As for Gram-negative isolates, levamisole significantly reduced (*p* < 0.05) the biomass of mature biofilms at only the two highest tested concentrations (9.37 and 18.75 mg/mL) when compared with the growth control (Figure 1c). *P. aeruginosa* isolates demonstrated a significant reduction in mature biofilm biomass at concentrations ranging from 1.17 to 18.75 mg/mL, as well as at 0.29 mg/mL (*p* < 0.05) (Figure 1e), while levamisole induced an increase in the biomass of *P. mirabilis* biofilms at concentration ranges of 0.03–4.68 mg/mL (*p* < 0.05) (Figure 1f). Confocal laser scanning microscopy revealed a reduction in live cells in mature biofilms of *S. pseudintermedius*, *P. mirabilis,* and *P. aeruginosa* and an increase in dead/damaged cells (Figure 2, and the COMSTAT™ analyses revealed an overall significant reduction (*p* < 0.01) in the maximum thickness of the biofilms (Figure 3e–h).

### 3.3. Effect of Levamisole on Bacterial Biofilm Growth

**Figure 4:**
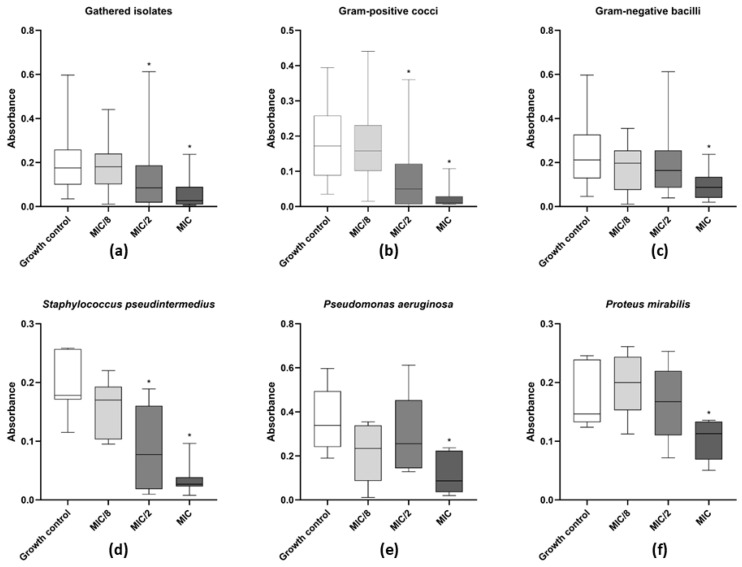
Activity of levamisole on growing biofilms of bacteria recovered from dogs with otitis externa. Data expressed as mean ± standard deviation of biomass absorbance after staining with crystal violet. (**a**) General profile of levamisole activity on growing biofilms of all tested isolates; (**b**,**c**) activity against Gram-positive cocci or Gram-negative bacilli, respectively; (**d**–**f**) activity against *Staphylococcus pseudintermedius* (*n* = 11), *Pseudomonas aeruginosa* (*n* = 11), and *Proteus mirabilis* (*n* = 12). * *p* < 0.05 indicates significant differences compared with untreated controls.

As for the effect of levamisole on biofilm formation by the 50 tested isolates, a significant reduction in biofilm biomass was observed at MIC and MIC/2 (*p* < 0.05) relative to the biofilm growth control (Figure 4a). Levamisole significantly reduced biofilm formation of *S. pseudintermedius* at both MIC and MIC/2 concentrations (*p* < 0.05) (Figure 4d) compared with the growth control.

As for Gram-negative bacterial isolates (Figure 4c), levamisole significantly reduced the biomass of growing biofilms of *P. mirabilis* (*p* < 0.05) (Figure 4e) and *P. aeruginosa* (*p* < 0.05) (Figure 4f) at MIC within the first 48 h of growth. Confocal laser scanning microscopy showed a great reduction in biofilm biomass and an increase in dead/damaged cells (Figure 2), and COMSTAT analyses revealed an overall reduction (*p* < 0.05) in the maximum thickness and biomass average thickness of growing biofilms and an increase (*p* < 0.01) in surface-to-volume ratio, indicating a loss in biofilm cohesion (Figure 3a–d).

**Figure 5:**
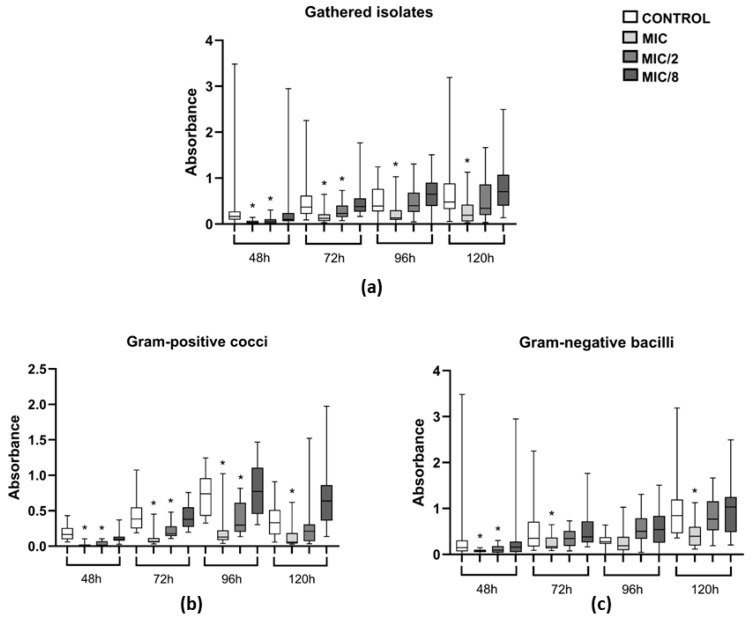
Evaluation of the activity of levamisole on growing biofilm and persistence of 12 bacterial isolates from dogs with otitis externa over 120 h of incubation. Data expressed as mean ± standard deviation of biomass absorbance measured by crystal violet. (**a**) General profile of levamisole activity on growing biofilms of the 12 tested isolates; (**b**,**c**) activity against Gram-positive cocci and Gram-negative bacilli, respectively. * *p* < 0.05 indicates significant differences compared with untreated controls.

Over a period of 120 h, levamisole at MIC significantly reduced the biomass of growing biofilms of the 12 tested isolates (3 *S. pseudintermedius*, 3 *S. schleiferi*, 3 *P. mirabilis*, and 3 *P. aeruginosa*) at all evaluated time points: 48, 72, 96, and 120 h (*p* < 0.05). When tested at MIC/2, levamisole significantly reduced biofilm biomass at 48 and 72 h (*p* < 0.05) (Figure 5a). As for Gram-positive isolates, levamisole led to significant reductions in biofilm biomass at both MIC and MIC/2 at 48, 72, and 96 h (*p* < 0.05), while at 120 h, levamisole reduced biofilm biomass at MIC only (Figure 5b). In contrast, levamisole induced biomass reduction in growing Gram-negative biofilms at MIC at 48, 72, and 120 h (*p* < 0.05) of growth, while at MIC/2, a significant reduction was observed only at 48 h of incubation (Figure 5c).

## 4. Discussion

Antimicrobial resistance represents a global public health challenge requiring integrated strategies across all sectors of human, animal, and environmental health to contain its spread, in line with the One Health approach. In veterinary medicine, due to the close relationship between humans and animals, a major concern is the potential transmission of resistant microorganisms from animals to humans, either as pathogens or as commensal organisms [26]. In this context, companion animals may act as disseminators of antimicrobial-resistant bacteria within household environments, including multidrug-resistant strains, posing a threat to public health [9,27]. Therefore, in the field of dermatology, diseases such as pyodermatitis and otitis are highly prevalent in clinical practice, with an increasing frequency in the recovery of multidrug-resistant isolates [8].

Costa et al. [28] argued that *Pseudomonas aeruginosa*, an opportunistic pathogen commonly associated with otitis externa and media, is frequently involved in cases of therapeutic failure and clinical relapse, which promotes the selection of multidrug-resistant strains including carbapenem-resistant isolates. In 2024, the World Health Organization (WHO) [29] released a priority list for the research and development of new therapeutic strategies aimed at controlling the emergence of antimicrobial resistance and multidrug-resistant strains, highlighting Enterobacterales as critical priority pathogens. This list also includes other bacteria classified as high-priority targets for drug development or repurposing, such as carbapenem-resistant *P. aeruginosa*, vancomycin-resistant *Enterococcus faecium,* and methicillin-resistant *Staphylococcus* species, including *S. pseudintermedius* (MRSP) and *S. schleiferi* (MRSS), which are also frequently associated with cases of canine otitis externa [4,8,30].

Due to difficulties in establishing the disease etiology and treatment, there has been an increase in cases of canine otitis externa associated with recurrent bacterial infections resulting in therapeutic failure and in the development of antimicrobial resistance. Farfán et al. [8] demonstrated that the use of antiseptic agents, such as ear cleansers, may cause dysbiosis, promoting the establishment of pathogenic microorganisms. In addition, perpetuating factors such as hyperplasia of the ear canal and bacterial infections in the middle and inner ear can contribute to the failure of topical therapy. Another critical factor contributing to the recurrence of otitis is the presence of biofilm-producing strains that maintain infection even after treatment, particularly among *Staphylococcus* and *Pseudomonas* species [6,15].

The most concerning cause leading to the emergence of antimicrobial resistance in otitis externa-associated bacteria is the challenge of treating this condition. The wide range of topical antimicrobial products commercially available and their indiscriminate use, especially regarding proper administration and treatment duration, are common shortcomings [6,31]. The frequent use of otological products in recurrent cases, particularly in biofilm-associated otitis, combined with the often-inappropriate use of broad-spectrum antibiotics, contributes to increased selective pressure, leading to colonization by resistant strains [28,30].

Therefore, there has been growing interest in the investigation of novel applications for non-antibiotic compounds as alternatives or adjuvants of topical antimicrobial and antibiofilm therapies for canine otitis externa. Previous studies have reported antibiofilm activity of compounds such as N-acetylcysteine and narasin in combination with antimicrobial agents [5]. Regarding *Staphylococcus aureus* isolates, compounds such as narasin combined with EDTA and bis(pyrazolyl)methane have shown promising results in the treatment of bacterial otitis [4,32]. In addition, studies have indicated that clove essential oil (*Syzygium aromaticum*) and narasin combined with EDTA exhibit antimicrobial activity against *Pseudomonas aeruginosa* strains [4,28].

In this context, the study investigated the antimicrobial and antibiofilm potential of levamisole against bacteria recovered from recurrent otitis externa in dogs. Levamisole is a drug approved for veterinary use and recognized for its immunomodulatory activity in human medicine. Levamisole MICs ranged from 0.58 to 2.34 mg/mL. Similar data have been reported in previous studies, which observed levamisole MIC values of 0.512 and 2.048 mg/mL against *P. aeruginosa* and *A. baumannii* strains, respectively [21]. Moreover, the MIC ranges against Gram-negative bacteria were higher than those against Gram-positive ones. This may be attributed to the more complex cell wall structure of Gram-negative bacteria compared with that of Gram-positive bacteria [23], which may reduce the ability of levamisole to penetrate Gram-negative cells.

Few studies have investigated the antimicrobial and antibiofilm activity of levamisole. Brilhante et al. [20] demonstrated its antifungal potential against *Coccidioides posadasii* and *Histoplasma capsulatum*, while Seleem et al. [21] reported the use of levamisole as a compound with antimicrobial and antibiofilm activity against *Acinetobacter baumannii* and *Pseudomonas aeruginosa* strains. In addition, experimental studies have demonstrated that dietary levamisole enhances immune responses and increases protection against *P. aeruginosa* infection in fish, suggesting a potential indirect antimicrobial activity through immunomodulation [33]. Regarding its activity against yeasts, Srilkala et al. [34] reported the use of levamisole as an adjuvant agent for the treatment of canine *Malassezia* spp. infections in combination with ketoconazole, exploring its immunomodulatory potential. However, further studies are required to support its use as an antimicrobial agent, since the yeasts of the genus *Malassezia* spp. are also cited as secondary agents associated with canine otitis externa [6].

Levamisole has demonstrated therapeutic efficacy in both humans and various animal species, including poultry, horses, and sheep, with well-documented anthelmintic, immunomodulatory (as an adjuvant), and anti-inflammatory properties [35]. Levamisole is well known for its anthelmintic effects, particularly for the control of gastrointestinal nematodes in farm animals [36], which it achieves through the selective activation of cholinergic ion channel receptors, leading to initial muscle contraction followed by neuromuscular paralysis in the parasite [37]. In the mammalian immune system, levamisole enhances T lymphocyte and macrophage activity and is especially used in the treatment of neglected tropical diseases in humans [19,38]. In rats, it has also demonstrated stress-modulating effects by reducing plasma cortisol and free radical levels [39].

Considering that biofilms of microorganisms commonly isolated from dogs with otitis externa play an important role in the pathogenesis of otitis due to their reduced susceptibility to antimicrobials, the antibiofilm activity of levamisole was investigated. In the present study, levamisole was initially assessed against mature biofilms to evaluate its ability to disrupt this bacterial structure. Levamisole eradicated mature biofilms of 50 bacterial isolates, with MBECs ranging from 4.68 to > 18.75 mg/mL, which were significantly higher than the MICs. This occurs because bacteria in biofilms exhibit greater resistance to antimicrobials and other chemical stressors compared with their planktonic counterparts [40].

The susceptibility to levamisole differed when comparing planktonic to biofilm growth, mainly due to biofilm structure and differences in gene expression. Biofilm-forming bacteria exhibit distinct gene expressions compared with their planktonic counterparts. Furthermore, the biofilm polymeric extracellular matrix limits the penetration of substances, which is another important factor for their persistence in the environment [41].

Previous studies have reported the effect of levamisole as an inhibitor of *Quorum sensing*, which is a communication system in bacterial communities coordinating the formation, maturation, and dispersal of biofilms as well as the exchange of virulence genes between resident microorganisms [21,40]. These data were corroborated by the confocal laser scanning analyses, which showed that mature biofilms exposed to levamisole at MBEC were thinner, less robust, and presented a greater number of dead/damaged cells.

In addition to interfering with biofilm viability, levamisole reduced the biomass of mature biofilms. Overall, it was observed that, for Gram-positive cocci, concentrations below the IC50 were sufficient to reduce mature biofilm biomass, demonstrating a better response. In contrast, Gram-negative bacilli required higher concentrations of the drug to achieve a similar effect, exceeding the cytotoxicity threshold.

However, given the potential cytotoxicity associated with the concentrations required to achieve an effect against mature biofilms, it is hypothesized that levamisole may be effective at lower doses. This hypothesis is supported by levamisole’s ability to disrupt biofilm architecture, suggesting that, when used in combination with conventional antimicrobial agents, it could enhance their therapeutic efficacy. Such an approach is particularly relevant in cases of otitis externa involving biofilms composed of both Gram-positive and Gram-negative bacteria [42]. In this context, the use of levamisole at reduced concentrations combined with classic antimicrobials may represent a promising antibiofilm strategy for future therapeutic applications.

The activity of levamisole on biofilm formation and maintenance, as well as on the dynamics of biofilm formation over 120 h, was analyzed to determine its applicability as a method of preventing and controlling biofilm growth in canine otitis externa. A general reduction in the biomass of growing biofilms was observed at levamisole MIC (1.17 mg/mL) at all analyzed time points (48, 72, 96, and 120 h) for all studied isolates. Furthermore, levamisole proved effective in inhibiting the growth of Gram-positive cocci biofilms over 96 h at a subinhibitory concentration. Confocal laser scanning analysis showed that biofilms grown in the presence of levamisole at MIC were thinner, less robust, and composed mainly of dead/damaged cells.

Considering that no previous studies have investigated the effect of levamisole on growing biofilms, it is essential to evaluate whether levamisole can interfere during the various stages of biofilm formation. One hypothesis to be explored is its potential effect on the initial adhesion of bacterial cells to biotic surfaces, a critical step in biofilm establishment and maturation [41].

The results of this study showed that levamisole inhibits biofilm growth for up to 120 h, supporting sustained antibiofilm strategies previously demonstrated in ex vivo porcine skin models [43]. These findings reinforce the potential of levamisole as a long-term approach to preventing biofilm formation in otitis externa.

Concerning the cytotoxicity test, the modal MIC found in this study was lower than the IC50 of levamisole (1.2 mg/mL) against the MRK5 cell line. However, before performing in vivo clinical trials, further cytotoxicity assays using other cell lines representative of the ear canal epithelium, such as keratinocytes, are necessary. Levamisole is known to have a narrow therapeutic window when administered systemically [19,44]. In contrast, in the context of canine otitis externa, it is proposed for topical use, and no reports have been found regarding systemic effects following topical application.

Considering the emergence of resistant strains within the One Health framework, it is important to emphasize the need for drugs that are not associated with antimicrobial resistance and that exhibit antibiofilm activity. Levamisole appears to be a promising strategy as a maintenance therapy for preventing recurrent bacterial otitis due to its ability to disrupt the biofilm structure.

Hence, this study represents a preliminary investigation into a new therapeutic perspective for the control and prevention of canine otitis externa. New in vitro research with polymicrobial biofilms is needed to better simulate the microbial complexity of infections combined with in vivo studies to validate clinical use.

## 5. Conclusions

This pilot study evaluated the in vitro antimicrobial and antibiofilm activity of levamisole against bacterial strains isolated from dogs with recurrent otitis externa. The findings indicate that levamisole showed antimicrobial activity against planktonic cells, reduced and eradicated the biomass of mature biofilms, and inhibited biofilm formation over 120 h. These results support the use of non-antibiotic compounds and drug repurposing strategies to prevent the development of antimicrobial resistance. Furthermore, the data encourage further research to evaluate the efficacy of levamisole in combination with conventional antimicrobials with the aim of enhancing penetration and activity against biofilm formation. Overall, the findings suggest that levamisole represents a promising approach as a maintenance therapy for the long-term prevention of biofilm formation, particularly due to its potential to disrupt biofilms and enhance treatment efficacy.

## 6. Patents

A patent was deposited encompassing the use of levamisole as a topical agent for the control and treatment of canine otitis externa (INPI BR 10 2024 019429 2).

## Figures and Tables

**Table 1:** Bacterial species, antimicrobial susceptibility profiles, resistance patterns, and biofilm-forming capacity of strains isolated from canine otitis externa.

Species	N	Resistance ^a^	MDR	Drugs	Biofilm-Forming Ability
*Staphylococcus pseudintermedius* (*n* = 11)	5	10–13	Yes	OXA *, CDM, CIP, ERI, GEN, LVX, STX, TET	Moderate
	3	5–7	Yes	CDM, CIP, ERI, GEN, LVX, STX, TET	Strong/Moderate
	3	1–2	No	AMP, CDM, STX	Strong/Moderate
*Staphylococcus schleiferi* (*n* = 3)	1	7	No	OXA *, CDM	Moderate
	2	0–1	No	CDM	Strong/Moderate
*Staphylococcus hominis* (*n* = 2)	1	1	No	CDM	Strong
	1	6	Yes	CDM, STX, TEI, VAN	Moderate
*Staphylococcus aureus* (*n* = 1)	1	5	Yes	CDM, CIP, LVX, STX, TET	Moderate
*Staphylococcus capitis* (*n* = 1)	1	1	No	CDM	Strong
*Staphylococcus cohnii* (*n* = 1)	1	1	No	CDM	Strong
*Staphylococcus auricularis* (*n* = 1)	1	6	Yes	CDM, LVX, STX, VAN	Weak
*Staphylococcus epidermitis* (*n* = 1)	1	13	Yes	AMO/SUL, AMP, AMC, CAX, CDM, CIP, DAP, ERI, GEN, LVX, LNZ, OXA, PEN, RIF, STX, TEI, TET, VAN	Moderate
*Enterococcus faecalis* (*n* = 3)	2	2	No	TET	Strong/Moderate
	1	4	Yes	ERI, STR, TET	Strong
*Proteus mirabilis* (*n* = 12)	6	0	No	-	Weak/Moderate/Strong
	1	8	No	AMP, AMC, FEP, ETP, MER, PRL/TAZ, PRL	Moderate
	2	4	Yes	AMP, FOS, PRL, STX	Moderate
	3	2–3	No	AMP, FOS, PRL, STX	Moderate/Strong
*Pseudomonas aeruginosa* (*n* = 11)	6	0	No	-	Weak/Moderate/Strong
	5	1–2	No	GEN, IMI, LVX	Moderate/Strong
*Escherichia coli* (*n* = 3)	2	0	No	-	Weak
	1	8	Yes	AMP, AMC, AZT, CAX, CIP, CEFU, PRL, STX	Weak

^a^ indicates the number of drugs to which the strains are resistant. MDR: multidrug resistance. * Oxacillin resistance indicates resistance to most ß-lactams. Abbreviations: AMP—Ampicillin; AMC—Amoxicillin–clavulanate; AZT—Aztreonam; CAX—Ceftriaxone; CEFU—Cefuroxime; CIP—Ciprofloxacin; CDM—Clindamycin; DAP—Daptomycin; ERI—Erythromycin; ETP—Ertapenem; FEP—Cefepime; FOS—Fosfomycin; GEN—Gentamicin; IMI—Imipenem; LNZ—Linezolid; LVX—Levofloxacin; MER—Meropenem; OXA—Oxacillin; PRL—Piperacillin; PRL/TAZ—Piperacillin–tazobactam; RIF—Rifampin; STX—Sulfamethoxazole–trimethoprim; TEI—Teicoplanin; TET—Tetracycline; VAN—Vancomycin; STR—Streptomycin.

**Table 2:** Planktonic and biofilm susceptibility profile to levamisole against bacterial isolates from canine otitis externa.

Species (*n*) *	MIC (mg/mL)	MBIC (mg/mL)	MBEC (mg/mL)
*Staphylococcus pseudintermedius* (11)	1.17 (5)	2.34 (3)	4.68 (1)
	0.58 (6)	4.68 (2)	9.37 (1)
		9.37 (1)	18.75 (2)
		18.75 (5)	>18.75 (7)
*Staphylococcus schleiferi* (3)	1.17 (3)	2.34 (2)	4.68 (2)
		4.68 (1)	9.37 (1)
*Staphylococcus hominis* (2)	1.17 (2)	2.34 (1)	4.68 (1)
		18.75 (1)	>18.75 (1)
*Staphylococcus aureus* (1)	0.58	2.34	>18.75
*Staphylococcus capitis* (1)	0.58	9.37	18.75
*Staphylococcus cohnii* (1)	1.17	9.37	18.75
*Staphylococcus auricularis* (1)	0.58	18.75	>18.75
*Staphylococcus epidermitis* (1)	1.17	9.37	18.75
*Enterococcus faecalis* (3)	2.34 (3)	4.68 (1)	18.75 (2)
		9.37 (2)	>18.75 (1)
*Proteus mirabilis* (12)	1.17 (3)	2.34 (1)	4.68 (1)
	2.34 (9)	4.68 (2)	9.37 (1)
		9.37 (8)	18.75 (6)
		18.75 (1)	>18.75 (4)
*Pseudomonas aeruginosa* (11)	1.17 (2)	4.68 (4)	>18.75 (11)
	2.34 (9)	9.37 (4)	
		18.75 (3)	
*Escherichia coli* (3)	1.17 (3)	4.68 (3)	9.37 (1)
			>18.75 (2)

* Numbers of isolates are shown in parentheses. MIC: minimum inhibitory concentration. MBIC: minimum biofilm inhibitory concentration. MBEC: minimum biofilm eradication concentration.

## Data Availability

The raw data supporting the conclusions of this article will be made available by the authors on request.
